# Microwaves in the cold war: the Moscow embassy study and its interpretation. Review of a retrospective cohort study

**DOI:** 10.1186/1476-069X-11-85

**Published:** 2012-11-14

**Authors:** J Mark Elwood

**Affiliations:** 1 Epidemiology & Biostatistics, School of Population Health, The University of Auckland, Private Bag 92019, Auckland, Mail Centre 1142, New Zealand

**Keywords:** Radiofrequencies, Cancer, Health, Cohort study

## Abstract

**Background:**

From 1953 to 1976, beams of microwaves of 2.5 to 4.0 GHz were aimed at the US embassy building in Moscow. An extensive study investigated the health of embassy staff and their families, comparing Moscow embassy staff with staff in other Eastern European US embassies. The resulting large report has never been published in peer reviewed literature.

**Methods:**

The original report and other published comments or extracts from the report were reviewed.

**Results:**

The extensive study reports on mortality and morbidity, recorded on medical records and by regular examinations, and on self-reported symptoms. Exposure levels were low, but similar or greater than present-day exposures to radiofrequencies sources such as cell phone base stations. The conclusions were that no adverse health effects of the radiation were shown. The study validity depends on the assumption that staff at the other embassies were not exposed to similar radiofrequencies. This has been questioned, and other interpretations of the data have been presented.

**Conclusions:**

The conclusions of the original report are supported. Contrary conclusions given in some other reports are due to misinterpretation of the results.

## Background

Beams of microwaves from Soviet sources aimed at the US embassy building in Moscow were detected since 1953, increasing in intensity in 1975. In 1976, an ambitious epidemiological study was commissioned by the U.S. Department of State to investigate possible health effects on the staff of the US embassy in Moscow and their families. The study was carried out by Abraham Lilienfeld (deceased, 1984) and colleagues at the Department of Epidemiology at Johns Hopkins University. The study has never been published in detail. It has been cited several times, with varying interpretations. This review is based on the main 1978 report [1], obtained from the Johns Hopkins University library, and on published literature referring to it. The frequency was 2.5 to 4.0 GHz and the exposure levels, while low compared to accepted exposure standards, were higher than typical present-day exposures of the public to, for example, cell phone base stations, so the study has relevance to current issues of health effects.

### Methods for this review

The original report was obtained from the Johns Hopkins University library ( https://catalyst.library.jhu.edu/). Searches for peer-reviewed material reporting on the study were carried out using PubMed, citation indexes, and major reports and reviews on health effects of radiofrequencies, up to September 2011. A senior living author of the original report was also contacted to identify any other sources; but was not involved in this review. General media coverage and ‘grey’ literature could not be comprehensively reviewed, and so is not included.

### Review - study design

The key aspect of Lilienfeld’s study design was to compare the Moscow embassy staff and their dependents with the staff and dependents at other eastern European U.S. embassies, who would have had similar selection procedures, and many similarities in their work and lifestyle. In this retrospective cohort study, the exposed group were staff who had served in the Moscow embassy during the period January 1 1953 to June 30 1976, and their dependents who lived in Moscow; and the comparison group were staff who served in other selected Eastern European embassies or consulates during the same period of time, and their dependents; in Belgrade, Bucharest, Budapest, Leningrad, Prague, Sofia, Warsaw, and Zagreb. These posts were chosen for their general similarity to Moscow in climate, diet, geographical location, disease problems, and general social milieu. Individuals who served in both Moscow and one of the comparison posts were counted in the Moscow group.

### Exposures to radiofrequencies

Exposure information is given in the main report and an appendix, and in more detail in a further assessment published later [2]. There was nearly continuous monitoring in the Moscow embassy from early 1963, and monitoring of other buildings further from the embassy at least every few months, but for earlier periods measurements were sparse. Tests for microwave radiation (0.5 Ghz to 10 Ghz) at the other embassies chosen were made periodically, at least once or twice a year but up to several times per month, and “only background levels have been detected at these Eastern European embassies” (page 3).

From 1953 to May 1975, the microwave beam came from a source in a Soviet apartment building about 100 m west of the 10 floor embassy building, affecting the west facade of the central building, with highest intensities between the third and eighth floors. The frequency was from 2.5 to 4.0 GHz and maximum exposures are given as up to 5 μW/cm^2^, 9 hours per day [1]. However Appendix 11 notes: "In general, individual exposures would have been much less than the maximums because of location away from a window or movement to other rooms or floors and the fact that some hours of signal operation were at night. ‘ Background’ levels existing when signals were off would be lower than the maximum signal levels by at least a factor of one thousand”. In the later report, the average power density in the rooms exposed is estimated as 1.5 μW/cm^2^[2]. Living quarters for employees and dependents were on the third to seventh floors, and similar levels were estimated for the kitchens and bedrooms, but living rooms, dining areas and bathrooms were not exposed.

From May 1975 there were beams from two sources, originating from buildings about 100 m east, and south, of the embassy. Maximum exposures are given as up to 15 μW/cm^2^ for 18 hours a day [1]. In the later report [2], the highest average levels given are 10.2 μW/cm^2^ in a 10th floor room, with average levels of exposure of staff of from 1.3 to 3.3 μW/cm^2^. The highest reading recorded was 24 μW/cm^2^ close to a window in a 10th floor room for a two hour period. On 6 February 1976, screening was installed on windows, which along with reductions in transmitter power, reduced the levels in even directly exposed rooms to less than 0.1 μW/cm^2.^

Estimates were made of the actual number of people working or living in exposed areas [2]. From 1953 to 1976 there were in total 1827 employees, whose tours of duty were usually two years, and about 3000 dependents; there were about 240 employees exposed to levels of 1.5 μW/cm^2^ for approximately 2 hours during the workday. There were 15 residential apartments, and about 660 residents (staff, dependent adults and dependent children) were exposed, with again average exposures of around 1.5 μW/cm^2.^ From May 1975 to Feb 1976, there were 26 employees with exposures of 1.5 μW/cm^2^ for 4 to 8 hours during work days. During this period the beam was more sharply focused on the upper floors, and in the living quarters the exposures would not have exceeded 0.8 μW/cm^2^.

### Identification of subjects and health information

Identifying eligible employees and dependents and linkage to death certificates and information on morbidity was a huge task; up to 50 people were employed in abstracting and coding health records, which were held in several different places. For most personnel, there were six or seven medical examinations available, with the maximum exceeding 20. Psychiatric examinations were also abstracted when available. An abbreviated abstraction form was used for dependents under the age of 12. For quality control, 10% of medical records were independently abstracted by two people, and 5% of all abstracts were checked by the investigators (page 14). In addition, a comprehensive Health History Questionnaire (HHQ) was developed and sent to employees and dependents for self-completion. This produced an “unacceptable” response rate of some 30% (page 27), and therefore “an ambitious system of tracing and interviewing state department employees by telephone” was set up.

### Methods of analysis

As “hundreds of factors” were examined, the analyses assessed three logical consequences for any condition increased by radiofrequency exposures specific to the Moscow embassy:

(1) the condition would be more common in the Moscow group than in the comparison group,

(2) within the Moscow group, it would be more common in those with greater estimated exposure to radiofrequencies, and

(3) amongst the Moscow group it would be more common in those who had spent a longer time in Moscow.

For mortality, for each of the Moscow and the comparison groups, standardized mortality ratios (SMRs) were computed, the expected numbers being based on death rates for the U.S. population for 59 causes of death, by sex, 5 year age group, and 5 year calendar period, using Monson’s program [3]. For recent years, data for earlier available years was used. Exact 95% confidence limits were based a Poisson distribution for the observed numbers, assuming no variance in the expected numbers. The report says data for the U.S. ‘white’ population was used (page 42); there is no data given on race.

Morbidity data was obtained from abstracts of medical records and from the Health History Questionnaires, distinguishing between conditions ever present and those first present after arrival at the index post (Moscow or other). Annual rates of first occurrence per 1000 person-years were calculated and compared as standardized morbidity ratios (SMbR), after indirect standardization by year of entry and by age, each in 4 groups, giving 16 strata, by Breslow and Day’s method [4], with confidence limits as in mortality data. Although very many comparisons were made in this study, no formal methods to account for multiple testing were used.

### Results - tracing

4,388 employees were identified (Table 1); 1827 who had served in Moscow, and 2561 who had been only in the other postings. State department employees were easier to trace and had more complete information than non-state department employees. Although the report states that the distribution was similar for Moscow group and for the comparison group (page 67), 63% of the Moscow and 72% of the comparison group were state department staff, resulting in respectively 94 and 96% of staff being traced. Medical records were reviewed for 70% of Moscow staff and 77% of other embassies. The HHQ was sent to 94% of Moscow and 91% of other embassy staff. The response rate was higher for Moscow but was still low: 51% compared to 40% in the other embassy staff. 2819 adult dependents and 5474 children were identified who had lived at the posting, either in the embassy or consulate or in off-site accommodation.

**Table 1:** Employees and dependents identified, traced, and with health information

	**Total**	**Moscow**		**Other embassies**	
			%		%
employees identified	4388	1827		2561	
% State Department Employees			62.9		72.0
employees traced	4179	**1719**	94.1	**2460**	96.1
medical records reviewed (% of traced)	3094	1205	70.1	1889	76.8
number of examinations		8573		13137	
examinations per subject		7.1		7.0	
sent HHQ (% of traced)	3867	1622	94.4	2245	91.3
completed HHQ (% of traced)	1853	869	50.6	984	40.0
deaths (% of traced)	194	56	3.3	138	5.6
deaths with information (% of deaths)	181	53	94.6	128	92.8
Adult dependents identified	2819	1061		1758	
lived in embassy or consulate, %		436	41.1	797	45.3
Child dependents identified	5474	2161		3313	
lived in embassy or consulate, %		792	36.6	1285	38.8
HHQ = Health History Questionnaire					

#### Results - mortality

The mortality experience is described in detail, but as the employees were relatively young, the numbers are small (Table 2). The results are given comparing observed deaths with expected numbers based on US mortality data. For total deaths, the standardized mortality ratios (SMRs) compared to the US population were 0.47 for Moscow employees (total deaths 38 men, 11 women), and 0.59 for the comparison group (102 male deaths, 30 female). This was interpreted as a “healthy worker effect” and the ratios for heart disease were 0.49 and 0.38 respectively. Cancers are shown in more detail; the SMRs for all cancer were 0.89 for Moscow, and 1.1 for the comparison group. For brain tumors in the comparison postings, the SMR is significantly raised, based on 5 cases. The SMRs are increased for leukemia and for breast cancer both in Moscow and in the comparison embassies, but these are not statistically significant.

**Table 2:** Mortality in employees

	**Moscow**					**Other embassies**					**Ratio of SMRs**
	**obs**	**exp**	**SMR**	**95% CI**		**obs**	**exp**	**SMR**	**95% CI**		***added***
All causes	49	105.3	0.47	0.4-	0.6	132	223.7	0.59	0.5-	0.7	0.8
Arteriosclerotic heart disease	16	32.6	0.49	0.3-	0.8	28	73.2	0.38	0.2-	0.6	1.3
All malignant neoplasms	17	19.0	0.89	0.5-	1.4	47	41.1	1.1	0.8-	1.5	0.8
All malignant neoplasms - male	11		0.63			33		1.3			0.5
All malignant neoplasms - female	8		1.1			14		0.94			1.2
Digestive organs	3	4.6	0.65	0.1-	1.9	11	10.8	1	0.5-	1.8	
Brain tumours	0	0.9	0.0			5	1.5	3.3	1.1-	7.7	0.0
Lung	5	5.8	0.86	0.3-	2	11	12.2	0.90	0.4-	1.6	1.0
Leukaemia	2	0.8	2.5	0.3-	9	3	1.7	1.8	0.4-	5.3	1.4
Breast	2	0.5	4.0	0.5-	14.4	3	1.2	2.4	0.5-	7	1.7

obs = observed; exp = expected based on US mortality data; SMR = standardised mortality ratio; CI = confidence interval.

Also shown (numbers in Moscow, other embassies) : pancreas (1,1); Hodgkin disease (0,0); uterus (1,0); cervix (1,0).

Decimal places vary, but are given as in the original. Data from Table 5.6 in main report. Ratio of SMRs added by present author as an approximate measure.

The interpretation given is that “no differences were observed between the Moscow and comparison groups either in total mortality or in mortality from cancer” (page 243). It was noted that particularly for women the proportion of all deaths that were due to cancer was high, both in the Moscow (8 of 11 deaths) and in the comparison group (14 of 31); the authors state “however, it was not possible to find any satisfactory explanation for this, due mainly to the small numbers of deaths involved and the absence of information on many epidemiological characteristics that influence the occurrence of many types of malignant neoplasms.” (page 243).

The study also assessed mortality amongst dependents, both adults and children. Lower importance is given to these groups, as the information on exposures and on health records was less complete, although these limitations were “present to the same degree in both the Moscow and comparison groups” (page 243). It was concluded that “no differences in mortality were detected between the Moscow and comparison dependent groups of children or adults” (page 244).

#### Results - morbidity

The analysis was based on medical records and on the Health History Questionnaire. For several general indices, such as the number of examinations performed for a medical problem and the occurrence of a hospitalization or a medical evacuation, the Moscow and comparison groups were ‘virtually identical’ (page 104). The records gave reports of any significant medical problem, various specific findings such as increased blood pressure, the occurrence of 15 general medical conditions in men and 18 in women, and in both men and women, 70 diseases or medical conditions, abnormal evaluations in 19 body systems, and 44 selected medical conditions reported on examinations.

Morbidity results between the Moscow and the comparison postings groups were compared by standardized morbidity ratios (SMbR), based on the rates of first occurrence of each condition after the relevant posting, adjusted for age at entry and year of entry to the study, giving 16 strata, using a Poisson log linear model (p. 46–48). Of the 70 diseases listed, three in men showed significant differences (Table 3): venereal disease was more common in the Moscow group, and appendicitis and sleepwalking were more common in the comparison group. However, venereal disease was not more common in those who had served longer in Moscow. Eight other conditions in men and one condition in women were, but none of these was more common in the Moscow group than in the comparison group. The authors comment that those who had served longer were older, which would explain most of the differences seen. Similarly, three conditions in men and five in women were more common in those in Moscow who were exposed to higher levels of microwaves, but none of these was more common in the Moscow group than the comparison group, and only one, vaginal discharge in women, was associated both with length of time spent in Moscow and with assessed exposure.

**Table 3:** Frequencies of medical conditions and of symptoms, per 1000 person-years, after the first tour at the index post, for those conditions with statistically significant differences between employees serving in Moscow and in the comparison group

**Source of data**	**Conditions**	**Moscow**		**Comparison**			**Ratio of SMBRs**
		**Number**	**SMBR**	**Number**	**SMBR**	**P value**	***added***
***Medical records:***							
	General medical conditions*: no significant differences in 15 conditions in males, and 18 in females*						
	Diseases or conditions*; male employees*						
	Appendicitis	12	0.62	38.0	1.2	0.03	0.5
	Sleep walking	1	0.3	12	1.5	0.01	0.2
	Venereal disease	24	1.4	15	0.67	0.02	2.1
	*67 other conditions: no significant differences*						
	Diseases or conditions*; female employees; 70 conditions: no significant differences*						
	Abnormal evaluations *by body system: male employees; 19 body systems: no significant differences*						
	Abnormal evaluations *by body system: female employees; 19 body systems: no significant differences*						
	Medical conditions by disease group*; male employees*						
	Protozoal intestinal diseases	21	1.7	8.0	0.48	0.001	3.5
	Benign neoplasms	119	1.2	151	0.90	0.04	1.3
	Disease of nerves, peripheral ganglia	32	1.3	32.0	0.30	0.05	4.3
	Pneumonia	14	0.6	42	1.2	0.02	0.5
	*40 other conditions: no significant differences*						
	*Medical conditions by disease group; female employees*						
	Complications of pregnancy	11	1.7	9	0.67	0.04	2.5
	*43 other conditions: no significant differences*						
***Health history questionnaire:***							
	General conditions*: male employees*						
	eye problems	98	1.3	65	0.76	0.002	1.7
	psoriasis	12	1.7	3	0.37	0.009	4.6
	skin conditions	63	1.2	45	0.81	0.04	1.5
	*25 other medical conditions: no significant differences*						
	General conditions*: female employees*						
	eye problems	33	1.4	28	0.76	0.03	1.8
	anaemia	16	1.6	10	0.64	0.03	2.5
	ulcers	6	2.1	3	0.49	0.04	4.3
	*25 other medical conditions: no significant differences*						
	Symptoms*: male employees*						
	depression	38	1.3	22	0.73	0.004	1.8
	irritability	40	1.3	20	2.4	0.009	0.5
	difficulty in concentrating	36	1.4	12	0.52	0.001	2.7
	memory loss	29	1.6	11	0.50	0.008	3.2
	*16 other symptoms: no significant differences*						
	Symptoms*: female employees*						
	difficulty in concentrating	17	1.6	9	0.58	0.02	2.8
	other symptoms	13	1.8	6	0.51	0.01	3.5
	*18 other symptoms: no significant differences*						

*Ratio of SMbRs added by present author as an approximate measure*.

Of the 44 medical conditions, for men, three were significantly more common in the Moscow group (protozoal intestinal diseases, benign neoplasms, and diseases of nerves and peripheral ganglia), and one was significantly more common in the comparison group (pneumonia). Women showed a significant difference only in complications of pregnancy, more common in the Moscow group. None of these four conditions were associated with length of service or a higher level of assessed microwave exposure within the Moscow group.

Cancers are discussed in detail. The authors note that women had more cancers than men, and that the Moscow women more frequently reported multiple cancers. In men, the occurrence of ‘all cancer except skin cancer’ was more common amongst Moscow employees who were exposed, but was slightly lower in the Moscow group than in the comparison group.

Data from the HHQ represents the self-reporting of illnesses, and 28 specific medical conditions are assessed (Table 3). For men, three conditions were significantly more common in the Moscow group: eye problems (almost all refractive errors), psoriasis, and other skin conditions. None of these three was more common in those who had greater length of service or higher microwave exposure within the Moscow group. In the medical abstracts, refractive errors were the most commonly reported condition in both Moscow and the comparison groups, at very similar frequencies. In women the only significant differences were in eye problems, anemia, and ulcers, all more common in the Moscow group, but none of these were significantly increased in those with longer service or those more exposed to microwaves in Moscow.

Further questions dealt with the occurrence of symptoms, as distinct from defined health problems. The authors report “there was a clear pattern of a higher frequency of symptoms reported by the Moscow group than was reported by the comparison group” (page 156). For men, four groups of symptoms were more common in the Moscow group than in the comparison group (depression, irritability, difficulty in concentrating, and memory loss), but within the Moscow group all four of these symptoms were less common in the group exposed to microwaves than those unexposed or with uncertain exposure.

For women, again there was greater reporting of symptoms amongst the Moscow group, with two symptoms, difficulty in concentrating and an aggregate category of other symptoms, being significantly more common. Difficulty in concentrating was reported more frequently in the microwave exposed group within the Moscow group, although the difference was not statistically significant.

Medical conditions reported in the questionnaires were classified into the 44 categories used earlier. For men and for women, there were no significant differences. Total hospitalizations were less common in Moscow than in the comparison group; physician visits and accidents or injuries of any kind were of similar frequency.

For dependents, the likely exposures were lower and the information available on health records was more limited. In their overall summary, the investigators conclude based on medical records and health history questionnaires, amongst adult dependents, and for children, “the vast majority” of health problems were similar in Moscow and comparison groups. The only problem present to a greater extent in children who had lived in Moscow compared to the comparison group was the occurrence of mumps. There were no differences detected in the frequency of congenital anomalies.

### Summary of results as reported

In the investigators’ discussion (page 244) they emphasize that “literally hundreds of comparisons were made” and only two differences stood out from the medical record review, the increased rate of protozoal infections in Moscow male employees, and the slightly higher frequencies of most common kinds of health conditions in the Moscow group. There were greater differences in the self-reported data on the questionnaires, but there were no conditions which were more common in Moscow and showed a relationship to estimated microwave exposure or length of service within the Moscow group.

The overall conclusion of the investigators (page 246) was: “To summarize, with very few exceptions, an exhaustive comparison of the health status of the state and non-state department employees who had served in Moscow with those who had served in other Eastern European posts during the same period of time revealed no differences in health status as indicated by their mortality experience and a variety of morbidity measures. No convincing evidence was discovered that would directly implicate the exposure to microwave radiation experienced by the employees at the Moscow embassy in the causation of any adverse health effects as of the time of this analysis”.

### Reporting of this study in the literature

This study does not appear to have been published in any detail in a peer reviewed paper. One paper by Lilienfeld [5] describes the tracing methods and mortality results, but only for State Department employees. It also shows the results for conditions on medical abstracts which were increased with greater estimated exposure in Moscow, but as noted above, none of these were more common in Moscow than in the comparison postings. The paper discusses the problems of follow up, validity of exposure classification, and study power.

In 1979, in a symposium on health aspects of non-ionizing radiation at the New York Academy of Medicine, a presentation by Herbert Pollack, professor emeritus of clinical medicine at George Washington University (deceased, 1990) [6] opens intriguingly with “…after yesterday's discussions, it was evident that there is so much misunderstanding about the basic facts that I shall deviate from my prepared text and present a historical background to the so-called ‘Moscow’ situation…”. Pollack describes the exposures and the study results, and quotes the conclusions statement given above, emphasizing the comparison between the Moscow embassy staff and staff of other Eastern European embassies. Also at that symposium, Pollack says that the purpose of the microwave beams was unknown [7]. He claims that that some reports of microwave effects were either misquoted or invented in the media [8]. Pollack’s own role is not described, but Goldsmith [9] (see later) says he was the State Department Contract Officer. In a later article [10], Pollack comments on ‘false’ news stories that two U.S. ambassadors died of cancer after exposure to microwaves in Moscow; he states that the ambassadors’ office was on the unexposed side of the building.

The main publication of data from the study was by John Goldsmith (deceased, 1999), who gives a very different interpretation of the results. In a review of radiofrequency effects, which states "this article is an opinion piece, not an intended to be a balanced presentation of the literature" [11], he notes that “The most important comparison was with the employees of other Eastern European embassies and their dependents,” but continues “but it was not certain whether microwave exposures of the comparison group could also have occurred." (page 52).

Goldsmith presents a table of cancer mortality which emphasizes the comparisons between embassy staff and the general US population (Table 4), combining Moscow and the other embassies, and employees and dependents. Many of these comparisons show statistically significant differences. For example, for all cancers in employees and adult and child dependents, in Moscow and in other embassies combined, the observed and expected numbers were 116 and 80.5 (P<0.05). Goldsmith uses this as evidence that embassy staff and dependents in both Moscow and the other embassies were at increased risk, and all the embassies were exposed to microwaves; however he gives no other evidence for this. Goldsmith's figures show that the greater increase in total cancers was seen in dependents rather than employees, both in those who lived in the embassy and outside the embassy. In a similar way, combining Moscow and other embassies and combining employees and dependents, Goldsmith shows significant increases in deaths from leukemia, mainly in children, both those who lived in embassies and outside; in brain tumors, mainly in employees in the other European embassies; and in breast cancer, contributed to both by employees in Moscow and dependents in other embassies. He reports one particular result, the occurrence of multiple site cancers in Moscow, being 1.33 sites per person compared to 1.02 expected.

**Table 4:** Cancer mortality in employees and dependents, as given by Goldsmith

	**Moscow**			**Other embassies**			**Both groups**		
	**obs**	**exp**	**P**	**obs**	**exp**	**P**	**obs**	**exp**	**P**
***All cancers***									
Employees	17	19		47	41.1		64	60.10	
Dependents: lived in	5	1.5	*	14	5.5	*	19	7.00	*
Dependents: lived out	7	3	*	19	6.1	*	26	9.10	*
Children: lived in	2	0.5		1	1.3		3	1.80	
Children: lived out	2	0.83		2	1.7		4	2.53	
all children	4	1.33	*	3	3		7	4.33	
Totals	33	24.83		83	55.7	*	116	80.53	*
***Leukaemia***									
all employees and dependents	4	1.5	*	6	2.64	*	10	4.14	*
***Brain tumours***									
all employees and dependents	2	1.35		6	2.27		8	3.62	*
***Breast cancer***									
female employees and adult dependents	3	1.41		9	3.44		12	4.85	*

* P<0.05.

From Goldsmith ^11^ . The original also gives leukaemia, brain tumours, breast cancer subdivided as shown for all cancer.

Obs = observed; exp = expected.

In a further table Goldsmith gives his estimate of a positive association as being “convincing” in the Moscow study for red blood cell changes, white blood cell changes, and increased all-site cancer incidence for Moscow staff and other employees, and some other comparisons which depend on combining Moscow and other embassies. He cites the exposures of the Moscow embassy cohort as ranging from 5 to 18 μW/cm^2^ , giving these as estimated exposures rather than as maximum levels. Goldsmith’s conclusions, and comments on excesses of particular cancers, without giving confidence limits, are repeated in another paper [12].

In [9], Goldsmith presents results on complications of pregnancy, which shows an increased risk in Moscow compared with the other embassies after the initial tour of duty (P=0.04), which he interprets as relating to spontaneous abortion. As noted above, this was the only one of 44 medical conditions in women assessed from medical records that showed a significant difference. Goldsmith claims that a potential infertility effect in the study was modified before the final report. He gives data on leukemia deaths, derived from his earlier paper [11], but showing only employees and child dependents (which showed excesses over expected), and omitting adult dependents (which did not). Goldsmith states that [9] there was some evidence that employees in the other embassies were exposed as well, "but the contract officer dismissed the possibility as being based on hearsay".

In a more informal 1985 publication [13], Goldsmith says that his introduction to the subject was from a lawyer, relating the wife of a State Department employee, who had lived in the Moscow embassy building and had developed breast cancer. The employee produced some data on the numbers of cancer cases which Goldsmith compared to US general population data, and estimated a 6 to 8 times higher cancer incidence. At about this time the investigation by Lilienfeld and his team was announced. After the Lilienfeld report was published and Goldsmith made his initial interpretation that there was increased cancer in both Moscow and the other embassies, the lawyer provided him in 1979 with further material obtained from the State Department under the Freedom of Information Act, including data on some small studies of blood measures, a claim from an employee group that radiation exposures were occurring of other embassies, and claims that data on exposure and the occurrence of some cases of cancer was withheld from Lilienfeld's team, and that some of their findings were altered or deleted.

Goldsmith’s data have been cited in some influential reviews. The review of epidemiological studies for the International Commission on Non-Ionizing Radiation Protection (ICNIRP) [14] gives some results from the Moscow study attributed to ‘Lilienfeld cited by Goldsmith’ and noting that the source of the expected numbers is unclear, and also that it was unclear whether the other Eastern European embassies were exposed. The observed numbers given are those for the Moscow group only, but the expected numbers do not match those in Goldsmith’s table [11].

Rothman [15] gives for leukemia and brain cancer the relative risk (SMR) and confidence limits for Moscow embassy employees; the comparison to other embassies is not mentioned. Similarly, Moulder et al. [16] cite these same estimates, with the SMR for all cancer in Moscow employees only. Other papers briefly mention the Moscow study and report it as showing no increase in leukemia or brain cancer [17], cancers in general [16,18-20], non-Hodgkin lymphoma [21], or health conditions in general [18,20,22].

Johnston-Liakouris [23] claims that the Moscow study results support the existence of a “radiofrequency sickness syndrome”. In this paper, “the critical review of this study” by John Goldsmith is gratefully acknowledged. While no data are presented, it claims that the Moscow study shows statistically significant effects ‘relative to controls’ (without specifying which controls), for many effects. These include loss of appetite, which is given in the text of the main report as increased in the Moscow group, but this seems to be an error: the corresponding table does not show an excess.

Navarro et al. [24] state that the Moscow study showed elevated mutagenesis and carcinogenesis in US embassy employees in Moscow, and a dose–response relationship between various neurological symptoms and microwave exposure.

## Discussion

Is this a useful and relevant study? As a cohort study with multiple outcomes, a major limitation is the limited information on the exposure. The microwave exposures had a frequency range somewhat higher than most cell phone frequencies, and similar to microwave relays, radar, and satellite links [25]. The exposure intensities in Moscow were low, for example in comparison to the ICNIRP recommended limit for public exposure of 10,000 μW/cm^2^ for frequencies from 2 to 300 GHz [26,27], but the maximum exposure levels documented were high compared to typical levels of public exposure from all radiofrequency sources or from cell phone base stations. A 1980 paper gives a median exposure level of 0.005 μW/cm^2^ in US cities, and an estimate that 1% of the population were exposed to levels greater than 1 μW/cm^2^[28]. A more recent UK survey measured exposures from base stations and from all sources at 180 locations at 17 sites where people were concerned about their exposures [29]; exposures from base stations had a geometric mean of 0.003 μW/cm^2^ and 95% percentile of 0.07 μW/cm^2^. As such exposures have been the focus of much speculation and several studies of potential health effects [30-41], the results of the extensive Moscow studies are relevant in this context.

As discussed in the report (page 237) there were no available records showing where employees in Moscow had worked or lived. It was only possible to determine exposure status if the Health History Questionnaire had been returned and even so, many respondents could not remember the details of their work and living locations. Even when the data were available, the worksheets provided by the Department of State on exposures cover only two time periods, before and after May 1975, and it is stated that “the study staff was unable to get access to the basic data on the intensity measurements from which the worksheet was derived before the preparation of this report” (page 237). It is also noted that the highest levels documented were for a short period, from June 1975 to Feb 1976.

The possibility that one or more comparison posts were exposed to microwave surveillance is also discussed (page 238), and it is stated that “As far as could be determined, no microwave levels other than background intensities have ever been discovered. Unfortunately, no access to the underlying data collected was possible before the preparation of this report.” Goldsmith has concluded not only that the other embassies were exposed, but he assumes the exposures were similar to Moscow, as he combines the data from Moscow and the other embassies for his interpretation. There seems no direct evidence to support Goldsmith’s position.

If we accept the contrast between Moscow and the other embassies as valid, the study does provide much data; and with all this detail, there is no consistent pattern in regard to any particular disease.

The authors of the main report are appropriately cautious about the interpretation of their study, emphasizing its limitations. They conclude that “all that can be said at present is that no deleterious effects have been noted in the study population, based on the data that had been collected and analyzed” (page 246). They point out that the highest exposure to microwaves occurred during a short period from June 1975–1976, and there was only a short time from then until the end of the investigation for any effects to appear. They recommended that further assessments of the health of this group should be made in future years, and a surveillance system should be set up to monitor the occurrence of deaths and the proportion due to malignancies.

The study had multiple outcome variables, and no corrections for multiple testing were used. The authors instead emphasized the logical issue of looking for differences between Moscow and other embassies, and differences by estimated exposure and time spent in Moscow. For many outcomes, statistical power is low. This is discussed in the report and in a paper [5] giving minimum detectable risk ratios.

Some of the problems in interpretation may have arisen because although the objective of the study was to compare the disease experience of Moscow embassy staff with staff of other embassies, this comparison is not summarized numerically. The mortality tables give the observed and expected number of events and the SMRs compared to the general US population, separately for the Moscow embassy and the other European embassies. The morbidity tables similarly present SMbRs separately for the two groups. The interpretation depends on a subjective comparison of the SMRs or SMbRs between the Moscow and the other embassies groups, but the ratio of the SMR or SMbRs is not shown. This is almost certainly because SMRs are not mutually comparable as they are based on different standard populations. However, the effect is that even papers which correctly interpret the study as showing no increased risks do not present the relevant comparison in their extracted data; they may show the observed and expected numbers or the SMR in the Moscow group only, ignoring the comparison to other embassies, e.g. [15,16,19]. While a ratio of the SMRs or SMbRs is not correct, it should be a reasonable approximation as the age and sex distributions of the two embassy groups do not vary greatly, and so have been added here in Tables 2 and 3. The ratio will also be imprecise as the variance of each SMR is large. If direct standardization or other methods of control for age and sex differences had been used, and a ratio given, the study may have been less open to misinterpretation.

## Conclusions

The Moscow study was a major epidemiological study of radiofrequency exposure and deserves recognition as such. Much of the published commentary on the study is misleading. Perhaps the publication of this review will encourage those with direct knowledge of the study and its sequelae to contribute new information.

## Abbreviations

GHz: Gigahertz; μW/cm^2^: Microwatts per square centimeter; HHQ: Health History Questionnaire; SMR: Standardized mortality ratio; CI: Confidence interval; SMbR: Standardized morbidity ratio; Obs: Observed; Exp: Expected.

## Competing interests

The author declares that he has no competing interests.

## Author’s contribution

The named author reviewed the original report and the references given and prepared the manuscript.
